# Use of Gold Nanoparticles as Substrate for Diffusive Monitoring of Gaseous Mercury

**DOI:** 10.3390/ma11112119

**Published:** 2018-10-28

**Authors:** Paolo Papa, Ilaria Fratoddi, Iole Venditti, Francesca Vichi, Antonella Macagnano, Emiliano Zampetti, Andrea Bearzotti

**Affiliations:** 1 CNR, Institute of Atmospheric Pollution Research, V. Salaria, km 29.3, Monterotondo, 00015 Rome, Italy; vichi@iia.cnr.it (F.V.); a.macagnano@iia.cnr.it (A.M.); e.zampetti@iia.cnr.it (E.Z.); a.bearzotti@iia.cnr.it (A.B.); 2 Department of Chemistry, Sapienza University of Rome, P. le Aldo Moro 5, 00185 Rome, Italy; ilaria.fratoddi@uniroma1.it; 3 Department of Sciences, Roma Tre University of Rome, Via della Vasca Navale 79, 00100 Rome, Italy; iole.venditti@uniroma3.it

**Keywords:** gold nanoparticles, gaseous mercury, pollution

## Abstract

In the present work, the study and the performances of an adsorbent material for gaseous mercury employed in different diffusive bodies geometries is presented. The material is based on gold nanoparticles (AuNPs) deposited on quartz fibres filters, suitable for bonding the gaseous mercury through an amalgamation process. Following thermal desorption and analysis, the behavior of different diffusive samplers prototypes was compared. Both indoor and outdoor exposures were carried out in order to evaluate the advantages and shortcomings of the geometries in study at different sites. From the outdoor long-term exposures, a constant uptake rate (*U_r_*), with a low influence coming from the environmental conditions, was observed for the axial geometry, reporting a high coefficient of determination (R^2^ 0.97). Indoor exposures showed a higher reproducibility, along with a higher coefficient of determination (R^2^ 0.99). The presented results allowed us to observe different behaviors coming from two kinds of diffusive samplers designs, showing different adsorption rates and data dispersion. This allowed us to focalize our attention on the most suitable design from these two tested prototypes, for this kind of adsorbent material.

## 1. Introduction

Among heavy metals, harmful for human health, mercury has gained more attention and study in the last years [1,2,3]. As a pollutant, mercury can be found in water, soil and atmosphere. In the last case mercury can be present in three different forms: As gaseous elemental mercury, Hg° (GEM), gaseous oxidized mercury, Hg(II) (GOM) and particulate bounded mercury (PBM) [4,5,6]. All of these three species are generally indicated as total gaseous mercury (TGM) [7,8,9]. Gaseous elemental mercury (GEM), when present in its elemental state (Hg°), can persist in the atmosphere over a period of time from several months to more than one year, before being oxidized and removed by dry or wet depositions. This long permanence of GEM in the atmosphere gives it the time to distribute homogeneously, leading to a globally mean concentration with small differences. 

Currently the mean concentration ranges from 1.5–1.7 ng m^−3^ to 1.1–1.3 ng m^−3^, respectively in the northern and southern hemisphere [2,10]. 

Through a microbial process, mercury can be converted in methylmercury [CH_3_Hg]^+^, which is highly hazardous for human and wildlife health, as it is a toxic and persistent bio-accumulative substance [11,12]. 

The atmosphere is the most significant means for the diffusion of gaseous mercury, therefore monitoring atmospheric concentrations is important to understand its impact on the environment and its pollutions cycles [13,14]. 

At present, monitoring gaseous mercury relies mainly on the use of automated sampling systems. The operating principle of these instruments is mostly based on gold preconcentrators which, after a defined sampling time, are thermally treated in order to release the adsorbed mercury, successively quantified through Cold Vapor Atomic Absorption Spectroscopy (CVAAS) or Cold Vapor Atomic Fluorescence Spectroscopy (CVAFS) techniques [15]. The use of these instruments, along with benefits, as high accuracy and short term response, allowing a fine temporal resolution, presents also some drawbacks, such as: high costs associated with the instruments, skilled personnel for the use and maintenance, continuous power and gas cylinders supply [16].

As a consequence, research, especially in the last decades, has been focused on the development of new materials to be employed and alternative devices, easy to use, economic and flexible, with a wider possibility to monitor larger areas. 

The passive air samplers (PASs), usually used for persistent organic pollutants (POPs) [17,18,19] in long term monitoring campaigns, have been lately extensively investigated and applied to many environmental pollutants detection. Vapor mercury monitoring has also discovered a great interest in the use and development of PASs [20,21,22]. 

Among the new utilized materials, nanostructured ones stand out for use as gaseous mercury adsorbers; in fact, they can be able to interact and measure below ppb concentrations values [23,24,25]. Compared to active sampling systems, diffusive samplers can offer some advantages in handling due to the absence of power or gas cylinder supply, with low purchase costs. These characteristics can allow their application to gain a higher spatial sampling resolution, allowing a greater coverage to be achieved in the monitoring campaigns, making it possible to identify sources of pollution emissions spread over a large area.

On the other hand, compared to the active sampling systems, these devices present a lower accuracy and temporal resolution, due to their slow sampling process.

The theoretical basis of diffusive sampling can be found in expressions deriving from Fick’s first law of diffusion, relating the mass uptake by the sampler (*m*) to the diffusion coefficient of the target species sampled (*D_A_*), the gradient of concentration between the environment (*C*_0_) and next to the adsorbent (*C*_1_), the time of exposure (*t*), and the geometric characteristics of the sampler (cross-sectional area, A, and diffusion path length, *L*). In ideal conditions, when the adsorbent is a perfect sink, *C*_1_ can be assumed to be zero. Actually, a series of factors affect the diffusion process from the air onto the adsorbent, among them turbulence along the diffusion path, saturation of the adsorbent, back diffusion and pressure or temperature variations. The following expression, introducing a coefficient *k* to account for non-ideality:

(1) $$m=\frac{D_{A}\cdot A\cdot C_{0}\cdot t\cdot k}{L}\;$$

can be used. The uptake rate *U_r_* or sampling rate can be defined as:

(2) $$U_{r}=\frac{D_{A}\cdot A}{L}\;$$

Therefore, the environmental concentration (*C*_0_) in ideal conditions (when *k* equals 1) can be obtained by:

(3) $$\;C_{0}=\;\frac{m\;\cdot L}{D_{A}\;\cdot \;A\;\cdot \;t}=\;\frac{m}{U_{r\;}\cdot t}\;$$

In general, *k* depends on various factors among which the couple adsorbent/adsorbate and it is determined experimentally. 

The deviation of experimental uptake rates from the theoretical values is a good indication that the conditions of ideality are not met. According to the above equations, theoretical *U_r_* of 0.005 m^3^ day^−1^ and 0.075 m^3^ day^−1^ have been calculated, respectively referred to the axial and radial diffusive samplers. In this case the value of *k* for axial samplers is ~1 (near ideal behavior), whereas the radial shows a lower experimental uptake rate, as will be shown later.

The ideal adsorbent should be stable, reusable, selective and should not involve the build-up of equilibrium among gas-phase and adsorbed analytes. Therefore, the correct choice of the adsorbent is critical in determining the performance of the diffusive sampler, since it also affects the analysis techniques applied (solvent or thermal desorption). 

So far, in the field of diffusive samplers monitoring, different devices and sampling strategies have been developed, mostly based on the use of gold and silver (through the amalgamation process) or carbon sorbent materials [26,27,28]. Especially in the case of gold, the researchers are oriented in using nanostructured materials, exploiting the capacity of the higher surface area to volume ratio, with respect to conventional materials, aimed to increase the efficiency of the adsorption process, towards the TGM. The speciation of GEM vs GOM is difficult to achieve, as reported for active sampling equipment [19], and active sampling bias could also affect diffusive sampling results due to the calibration against these instruments usually performed in the development of passive devices. The most abundant fraction of gaseous mercury is GEM, nevertheless the amount of GOM could significantly vary according to the site and atmospheric conditions.

In this paper we report the results of a study regarding the development of a passive device for monitoring atmospheric TGM concentrations, based on gold nanoparticles [29], deposited on a quartz fibers support [30,31,32].

Preliminary trials to test the performances of the nanostructured material were carried out using different diffusive bodies. The prototypes were deployed both outdoors, where mercury ambient background concentrations could be found, as well as indoors. Two different geometries were tested, evaluating both their adsorption capacity and the relative sampling rate, when exposed in different conditions. Each sampler exhibited a characteristic SR. The relative results are presented and discussed.

## 2. Materials and Methods

### 2.1. Adsorbent Materials

Gold nanoparticles (AuNPs), utilized as adsorbent material, have been synthetized starting from the tetrachloroauric acid and following two well-established phases’ reduction procedure [33], optimized for dithiol ligands [34,35].

All the solvents and reagents used were purchased from Sigma-Aldrich (St. Louis, MO, USA): Tetrachloroauric (III) acid trihydrate (HAuCl_4_·3H_2_O), Tetraoctylammonium bromide (TOAB), Sodium borohydride (NaBH_4_), p-Terphenyl-4,4′′-dithiol (TR), Dichloromethane, Chloroform, Ethanol, Toluene, Petroleum Ether.

The so prepared AuNPs, were dispersed in dichloromethane solvent, with a final concentration of 1 mg/mL.

Quartz wool discs (Whatman^®^ quartz filters, Whatman plc, Maidstone, UK) consisting of quartz fibres (QF), 16 mm in diameter, 0.5 mm thickness, were used as supporting material, able to undergo continues high thermal desorption cycles. 

### 2.2. Instruments

A Tekran^®^ Model 2505 (Tekran Instruments Corporation, Toronto, ON, CA) mercury vapor calibration unit primary source, capable of generating defined selected quantities of vapor mercury was used for the calibration and control of the Tekran^®^ 2537A (Tekran Instruments Corporation) and to generate a defined gaseous mass of elemental mercury, was used to expose and test the adsorbent material capability. 

A Tekran^®^ Model 2537A ambient mercury vapor analyzer was used to measure the TGM environmental concentration, to be used as a reference for the devices in study, and to quantify the TGM desorbed by the samplers during their exposures.

An oven capable reaching a temperature of 500 °C, necessary for a fast and total desorption of the adsorbent material, has been used. 

### 2.3. Diffusive Samplers

**Figure 1:**
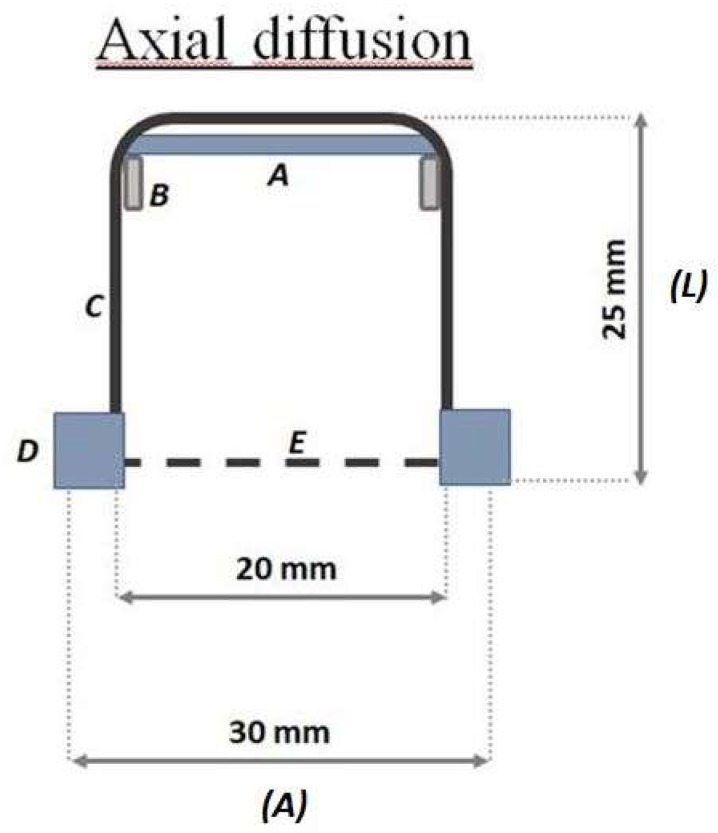
Schematic representation of the axial sampler. Adsorbent nanostructured material (A), teflon o-ring (B), glass cylinder (C), teflon cap (D) teflon net (E).

**Figure 2:**
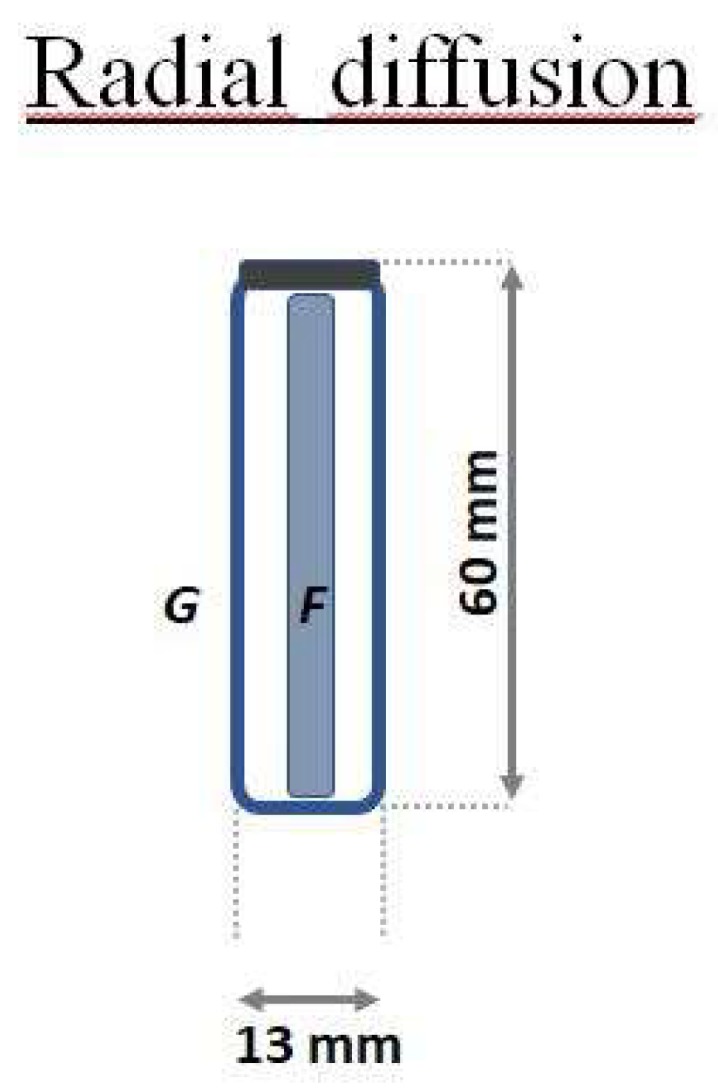
Schematic representation of a radial sampler. Adsorbent nanostructured material (F), the diffusive body (G).

**Figure 3:**
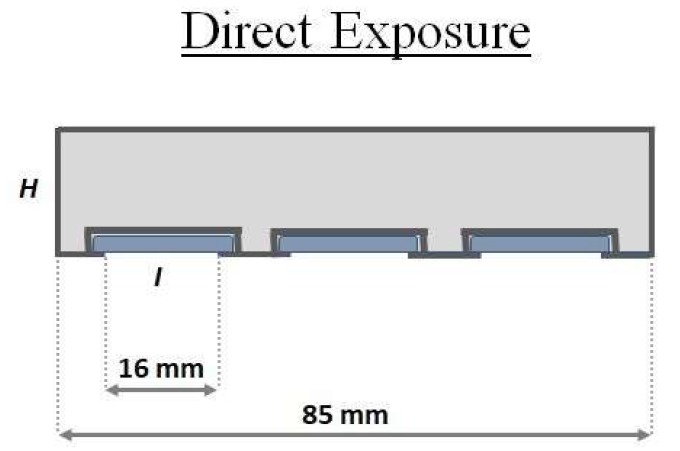
Schematic representation of the sampling grid. Structural holder (H), adsorbent nanostructured material (I).

The samplers geometries can be described as follows:

- A glass cylinder (inner diameter 20 mm, 25 mm in depth) with a cap and a diffusion teflon net (mesh 100 µm) (Figure 1), aimed to avoid undesired adsorption by using metallic nets.
- Three Radial diffusive bodies made of polyethylene (PE) with a microporous structure, 1.5 mm in thickness, an average porosity of 20 ± 5 μm, has been used for the radial diffusion sampling (Figure 2).
- A polystyrene (PS) holding grid, 85 mm × 40 mm, has been used to hold three samplers exposed directly to the ambient vapor mercury concentration, without any diffusion barrier (Figure 3).

### 2.4. Samplers Preparation

A solution volume of 50 μL, containing the AuNPs in dichloromethane, at a concentration of 1 mg/mL, was deposited on a thin disk of quartz wool fibers (QF) suitable as substrate support. The total active surface area, covered by the gold particles on each sample, was of 133 mm^2^. In this way, three circular samplers to be used in the axial geometry and three other circular samplers to be used for the direct exposure were prepared. All the samplers, based on the AuNPs, were prepared by drop casting deposition technique. In the case of the radial geometry, three different rectangular elongated quartz fibers forms have been used, so that they could be inserted inside the radial diffusive holder. The total active surface area of 133 mm^2^ deposited and the amount of 50 μL solution casted on the quartz fibers supports was the same for all the samplers (both for the circular and rectangular shape). Totally nine adsorption samplers, both with circular and rectangular shapes, have been prepared. This adsorbent material was previously studied, testing its adsorption capacity towards the gaseous mercury achieving good results, as reported in a precedent work [36]. In fact, it showed a high Hg adsorption capability, also when exposed to sub ppb concentrations, with a good stability and linearity. Moreover, when utilized in cyclic measurements, up to 35 times, the adsorbent material didn’t show any reduction in adsorption capability, allowing a further reuse.

### 2.5. Samplers Exposures

**Figure 4:**
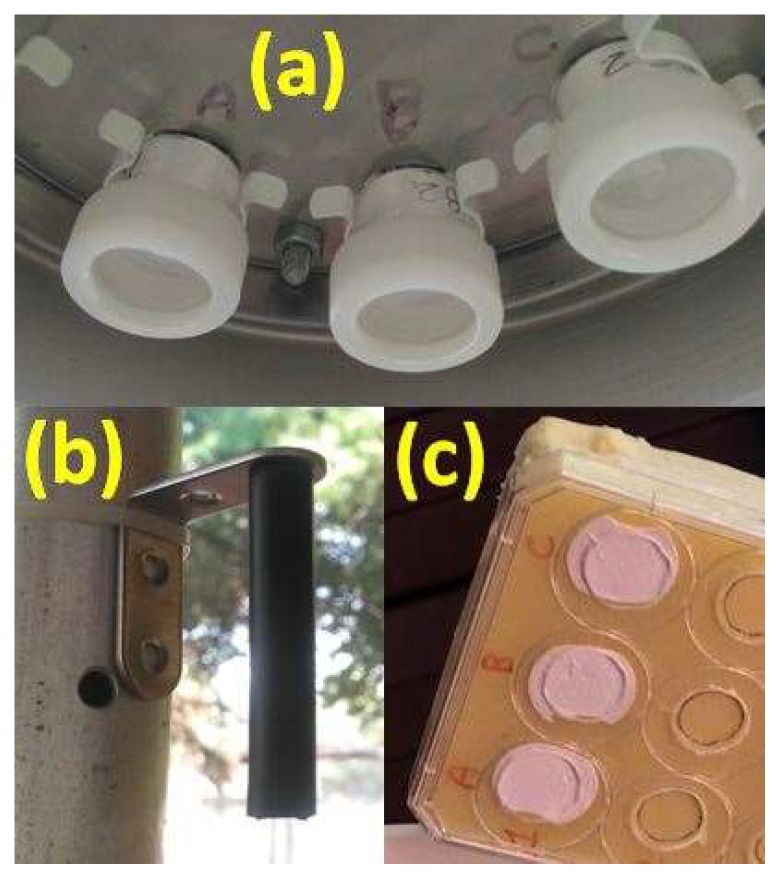
Outdoor exposure conditions, holders for: axial diffusive dosimeters (**a**), radial diffusive dosimeters (**b**), direct exposure (**c**).

The samplers were exposed to the outdoors in the countryside surrounding our laboratories to monitor mercury concentration. They were placed on a single main support, with few centimeters of distance between one another, to minimize the different conditions during the sampling period (Figure 4).

During sampling, the external gaseous mercury concentration was constantly monitored by the use of a Tekran^®^ 2537A, which recorded the data with an interval of 5 min. Calibrations and controls on Tekran^®^ 2537A have been performed both before, during and after the sampling campaign, to verify the correct calibration of the instrument, through the use of a Tekran^®^ Model 2505 Mercury Vapor Calibration Unit [37] and a suitable gastight syringe.

The data regarding the external vapor mercury concentration did not show any significant change during the sampling period, reporting a total mean concentration of 1.5 ± 0.5 ng m^−3^ of TGM.

The indoor measurements performed in a laboratory, have been done while avoiding the effects of turbulence, choosing the site in a way that the presence of personnel could not affect the sampling. In this case a continuous monitoring of gaseous mercury concentration was carried out by a Tekran^®^ 2537A as well; a mean concentration of 4.5 ± 0.5 ng m^−3^ was found.

Once the exposures were finished, the TGM adsorbed on the samplers have been thermally desorbed, placing them inside a hermetic quartz crucible and arranging it in an oven at a temperature of 500 °C. Under a synthetic air desorption flux, of 1 L min^−1^, the desorbed gaseous mercury was analyzed by a Tekran^®^ 2537A.

During the sampling exposure, the adsorption of the vapor mercury on the gold nanoparticles, takes place through an amalgamation process, as reported in literature [38,39]. In fact, gold is a well-known mercury adsorbent material, thanks to its strong affinity with mercury, leading to the formation of an amalgam [40].

Based on the results coming from this study, it has been possible to determine and associate a defined *U_r_* to each sampler, both for indoor and outdoor exposures.

## 3. Results and Discussions

### Sampling Rate

In this work an empirical *U_r_* was calculated according to the Equation (4):

(4) $$\;U_{r}=\;\frac{m}{C_{0}\cdot t}$$

The use of the axial geometry, where the adsorbent material is placed orthogonally to the incoming flow, on the bottom of a cylinder opened from one side (Figure 4a) either of a radial symmetry in which the gases enter through an elongated diffusion cylinder pores towards an adsorbing material placed inside (Figure 4b) leads to quite different results.

The calculation of the *U_r_* of these prototype samplers was possible by the use of a Tekran^®^ 2537A, measuring the vapor mercury concentration in the ambient (*C*_0_) and the mercury adsorbed on the samplers (m). All the measurements were always based on triplicate samplers, for each kind of exposure set up and each sampling period.

We started the experimental tests sampling in an indoor environment. The adsorbent materials were positioned in a laboratory room rarely visited, with no air turbulence and a mean gaseous mercury concentration of 4.5 ± 0.5 ng m^−3^ (temperature of ~22 °C, relative humidity of ~42% RH) sampling for a period of 31 days.

**Figure 5:**
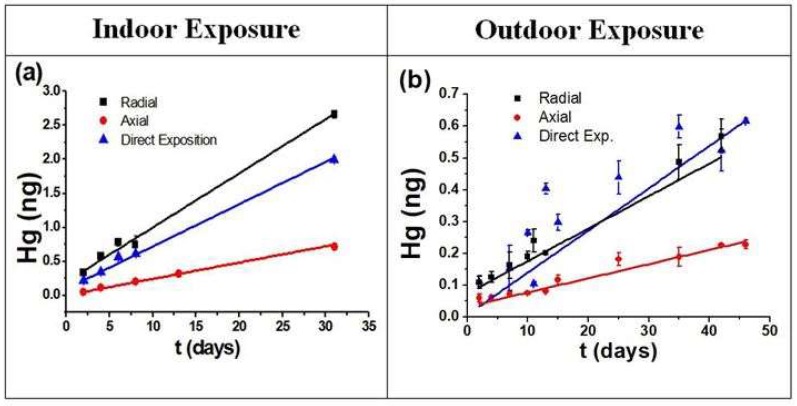
Adsorbed TGM on samples exposed indoors (**a**) and outdoors (**b**).

From the data reported in the Figure 5a it is possible to observe a different adsorption rate between the two samplers geometries, but all samplers show a straight linearity with a high reproducibility (the error bars, equal to one standard deviation, lie inside the marked point).

The adsorption linearities reported, could be explained by the absence of any air turbulence in the room and a stable vapor mercury concentration over time.

On the other hand, a different behavior was observed in the outdoor exposures, where the samples were exposed for a period of 46 days, at 1.5 ± 0.5 ng m^−3^ of vapor mercury concentration. The measurements have been repeated successively for shorter periods, after each desorption and measurement. During the outdoor exposition, the atmospheric conditions were constantly monitored. The average wind speed was of 0.9 m s^−1^, an average temperature of 17.3 °C was reported and a RH of ~48% was measured.

Finally the direct exposure (D.E.) of the adsorbent material, without any diffusion barrier at the ambient concentration was also carried out to account for the effects of an open air exposure on the surface (Figure 4c).

In Figure 5b data concerning the samplers exposed in outdoor conditions are reported. It is possible to note a larger dispersion of the data, especially for D.E.

The adsorption dispersion observed in the D.E. samplers, following the outside exposures (Figure 5b, blue mark), could be easily explained by the direct effects on the surfaces; it is not possible to define a real *U_r_* in this case.

**Figure 6:**
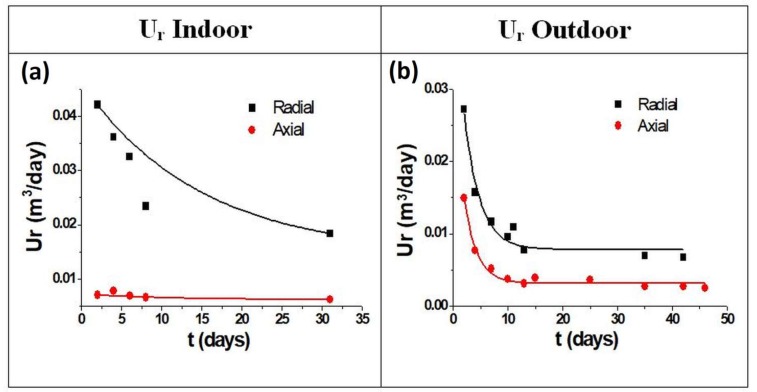
*U_r_* of the sample shelters following the indoor (**a**) and outdoor (**b**) exposures.

In Figure 6a,b differences in the *U_r_* are evident. In Figure 6a a slight decrease of the values is observed in the radial type SR, while a more stable and linear SR over the time was observed in the axial geometry.

A different SR behavior is observed in the outside exposures. As shown in Figure 6b, the uptake rate plots can be divided into two parts. A decreasing SR can be observed in the first days, followed by stabilization at lower values after the fifth day of exposure. These responses could be explained by the change of the concentration gradient into the diffusion gap during the first days, as also observed in previous studies on diffusive samplers [32,41]. The equilibrium concentration of the gaseous mercury upon the adsorbent material is, indeed, low at the beginning of sampling, resulting in a higher concentration gradient with a fast adsorption of the mercury compound. Since uptake is a cumulative process and uptake rate is relative to the entire sampling period, this should be extended so that the fast initial changes can be considered negligible. This is much more evident for the radial samplers, which are characterized by a shorter diffusion path and, hence, a faster sampling. Moreover, a greater dispersion is observed for the SR of radial samplers, while a lower and constant SR is reported for the axial ones, as summarized in Table 1.

The best results are obtained with the axial geometry. In fact, a higher coefficient of determination (R^2^) in both indoor and outdoor exposures was calculated for this geometry. From the data reported, it is possible to observe a good agreement between the theoretical and the experimental *U_r_* in the case of the axial sampler outdoors (0.005 m^3^ day^−1^), with a slight difference for indoors (0.006 m^3^ day^−1^). A different response is observed for the radial sampler, which, compared to a theoretical *U_r_* of 0.07 m^3^ day^−1^, showed an experimental *U_r_* of 0.03 m^3^ day^-1^ and 0.01 m^3^ day^−1^ for the indoor and outdoor expositions that were respectively observed.

## 4. Conclusions

The purpose of this work was to study the behavior of gold nanostructured adsorbent material in two different diffusive sampling geometries.

From the data reported (Figure 5a,b) a linear correlation between the adsorbed TGM and the deployment period, at a defined vapor mercury concentration, both outdoors and indoors, was observed.

In both cases, a different behavior in the adsorption rate of the samplers was evidenced.

In the outdoor exposures, the presence of a low vapor mercury concentration (1.5 ± 0.5 ng m^−3^) leads to a low *U_r_* of the samplers, which, associated with a major air turbulence, causes a high dispersion in the data, as indicated by a low coefficient of determination (R^2^), Table 1.

A different response was observed in the indoor exposures, where a higher gaseous mercury concentration (4.5 ± 0.5 ng m^−3^) led to a slightly greater *U_r_*.

Moreover, in the last case, the absence of any air turbulence generated a very low dispersion data, reporting a higher coefficient of determination (R^2^) for all the samplers, Table 1.

Following these measurements, we could evaluate a defined *U_r_* for each sampling shelter, with the relative advantages and shortcomings.

In particular, the best fit, both for the indoor and outdoor exposures, was observed using the axial sampler, for which a higher coefficient of determination (R^2^ 0.99–0.97 respectively) was found. Moreover, an agreement with the theoretical *U_r_* was observed, especially in the case of the outdoor expositions which totally fits (0.005 m^3^ day^−1^). Whereas, the radial samplers, especially when exposed in an outdoor environment (R^2^ 0.86–0.92 respectively), were characterized by a worse reproducibility, where the theoretical *U_r_* was also different in both indoor and outdoor expositions.

The axial geometry, coupled to the use of gold nanostructured materials, then seems to be the most reliable, with a low and constant *U_r_* over time and a low data dispersion for both indoor and outdoor exposures in vapor mercury detection.

According to these results, the presented axial geometry sampler showed a valid device in the detection of gaseous mercury for these kinds of adsorbent nanomaterial.

## Figures and Tables

**Table 1:** Indoor and outdoor SR, with the relative coefficient of determination R^2^.

Sampler	Indoor SR (m^3^ day^−1^)	Adj. R-Square	Outdoor SR (m^3^ day^−1^)	Adj. R-Square
**Axial**	0.006	0.99	0.005	0.97
**Radial**	0.030	0.99	0.010	0.92
